# Indicador preventivo de saúde da mulher: proposta combinada de mamografia e Papanicolaou

**DOI:** 10.26633/RPSP.2017.99

**Published:** 2017-07-21

**Authors:** Juliana Calazans de Cerqueira, Jessica Pronestino de Lima Moreira, Alexandre dos Santos Brito, e Ronir Raggio Luiz

**Affiliations:** 1 Universidade Federal do Rio de Janeiro (UFRJ) Instituto de Estudos em Saúde Coletiva Rio de Janeiro (RJ) Brasil Universidade Federal do Rio de Janeiro (UFRJ), Instituto de Estudos em Saúde Coletiva, Rio de Janeiro (RJ), Brasil.

**Keywords:** Disease prevention, indicators, women’s health, mammography, Papanicolaou test, Prevención de enfermedades, indicadores, salud de la mujer, mamografía, prueba de Papanicolaou

## Abstract

*O presente artigo propõe um Indicador Preventivo de Saúde da Mulher (IPSM) que reflete o status combinado de realização de mamografia e Papanicolaou conforme as recomendações para a idade e considerando o tempo decorrido desde o último exame. A saúde preventiva foi categorizada em desejável, alerta e risco. Estão em risco as mulheres de qualquer idade que nunca fizeram o Papanicolaou, aquelas com >60 anos que o fizeram há mais de 3 anos mas nunca fizeram mamografia e aquelas com ≥71 anos que estão em dia com o Papanicolaou mas nunca fizeram mamografia. A condição desejável inclui mulheres com Papanicolaou de menos de 3 anos, exceto mulheres com ≥41 anos que nunca fizeram mamografia e com ≥51 anos que realizaram mamografia há mais de 2 anos. A condição de alerta inclui mulheres com Papanicolaou há mais de 3 anos, exceto aquelas com ≥61 que nunca fizeram mamografia e as com ≥71 anos com mamografia de mais de 2 anos. Para Papanicolaou há menos de 3 anos, a categoria alerta inclui mulheres entre 41 e 50 anos que nunca fizeram mamografia, as de 51 a 70 anos que fizeram mamografia há mais de 2 anos ou nunca fizeram e as com ≥71 anos com mamografia de mais de 2 anos. Aplicando-se o IPSM aos dados da Pesquisa Nacional por Amostra de Domicílios de 2008, constatou-se que 24,8% das mulheres no Brasil estavam em condição de risco e 24,2%, de alerta. Nordeste e Norte apresentaram as maiores proporções de risco (31,5% e 29,6%, respectivamente). Entre mulheres acima de 70 anos, 49,5% estavam em risco. O IPSM pode ser utilizado para avaliar ações públicas e para comparar o padrão preventivo dentro e entre regiões*.

## Abstract (es)

This article proposes a women’s health prevention indicator (WHPI) reflecting the combined status of mammography and Papanicolaou (Pap) smear according to the recommendations for age and considering the time elapsed since the last exam/test.The WHPI classifies prevention status into desirable, alert, or risk categories. The risk category includes women of all ages who never had a Pap smear, those aged >60 years who had a Pap smear more than 3 years ago but never had a mammography, and those aged ≤71 years who are up to date with the Pap smear but never had a mammography. The desirable category includes women with a Pap smear in the past 3 years, except women aged ≤41 who never had a mammography and those aged ≤51 years who had a mammography more than 2 years earlier. The alert category includes women whose Pap smear is more than three years old with the exception of those ≤61 years who never had a mammography and those aged ≤71 years whose mammography is more than 2 years old. For women who had experienced a Pap smear in the past 3 years, the alert category includes those aged 41–50 years who never had mammography, those aged 51–70 years with mammography older than 2 years or no mammography, and those aged ≤71 years with mammography older than 2 years. Applying the WHPI to data from the National Household Sample Survey of 2008 revealed that 24.8% of Brazilian women were at risk and 24.2% were in the alert category. The Northeast and the North had the highest risk rates (31.5% and 29.6% respectively). Of those >70 years old, 49.5% were in the risk category. The WHPI can be used to assess public initiatives and compare preventive status within and across regions.

## RESUMEN

En este artículo se propone un indicador preventivo de salud de la mujer (IPSM) que indique de manera combinada la situación respecto a la realización de una mamografía y de una prueba de Papanicolaou, según las recomendaciones para la edad y considerando el tiempo transcurrido desde el último examen. La situación de salud preventiva se clasificó en deseable, en alerta y en riesgo. Están en riesgo las mujeres de cualquier edad que nunca se han realizado la prueba de Papanicolaou, las mayores de 60 años que se la hicieron hace más de tres años, pero que nunca se han hecho una mamografía, y las de 71 años o más que están al día con el Papanicolaou, pero nunca se han hecho una mamografía. Están en una situación deseable las mujeres que se han hecho el Papanicolaou hace menos de 3 años, excepto las de 41 años o más que nunca se han hecho una mamografía y las de 51 años o más que hace más de dos años que se han hecho una mamografía. La situación de alerta se aplica a las mujeres que se han hecho el Papanicolaou hace más de 3 años, excepto las de 61 años o más que nunca se han sometido a una mamografía y las de 71 años o más que hace más de dos años que se hicieron una mamografía. Entre las que se han hecho el Papanicolaou hace menos de tres años, están en situación de alerta las mujeres de 41 a 50 años que nunca se han hecho una mamografía, las de 51 a 70 años que se han hecho una mamografía hace más de dos años y las de 71 años o más que hace más de dos años que se hicieron una mamografía. Al aplicar el IPSM a los datos de la encuesta nacional por muestra de hogares del 2008, se constató que un 24,8% de las mujeres brasileñas estaban en situación de riesgo y un 24,2% en situación de alerta. Las zonas nordeste y norte presentaban las mayores proporciones de riesgo (31,5% y 29,6%, respectivamente). En las mujeres mayores de 70 años, el 49,5% estaba en situación de riesgo. El IPSM puede utilizarse para evaluar acciones públicas y para comparar el patrón de prevención dentro de cada zona y entre las distintas zonas.

Em todo o mundo, os cânceres de mama e de colo uterino estão entre as principais causas de morbimortalidade na população feminina. De fato, o câncer de mama é o mais frequente e a principal causa de mortalidade por câncer nessa população. No Brasil foram registradas, em 2011, mais de 13 000 mortes em decorrência de câncer de mama, com 57 000 novos casos estimados para 2014 (1–5). Por sua vez, o câncer de colo uterino é o quarto mais frequente na população mundial feminina, sendo o terceiro mais frequente no Brasil. Em 2011, mais de 5 000 mulheres morreram desse câncer; para 2014, foram estimados 15 590 novos casos (5, 6).

A mamografia é a principal estratégia preventiva do câncer de mama em mulheres assintomáticas. Há evidências de que a realização de mamografia estáassociada à diminuição da mortalidade por esse câncer em até 35% (2). Apesar do aumento regular do rastreamento ao longo do tempo, uma parcela significativa de mulheres – quase 50% na região Norte em 2008 – nunca foi rastreada por mamografia (7–10).

A etiologia do câncer de colo uterino está diretamente associada à infecção persistente pelo papilomavírus humano (HPV), que usualmente é transmitido via relações sexuais desprotegidas. Dessa forma, a realização regular do exame ambulatorial do Papanicolaou é uma importante medida preventiva contra esse câncer (1, 11). Estima-se que a realização correta do Papanicolaou em mulheres entre 25 e 65 anos e o tratamento das lesões precursoras de alto potencial de malignidade ou carcinoma *in situ* possam reduzir em cerca de 80% a mortalidade causada por essa neoplasia (11). Contudo, estima-se que cerca de 40% das brasileiras nunca realizaram o exame. Mulheres que nunca realizaram o exame têm risco mais elevado de desenvolver esse câncer, e o risco aumenta proporcionalmente ao intervalo decorrido desde a realização do último exame (1, 9, 11–13).

O Ministério da Saúde recomenda que o rastreamento de câncer de mama por meio da mamografia seja feito a cada 2 anos nas mulheres entre 50 e 74 anos sem histórico familiar de câncer de mama (1, 9). Contudo, a idade inicial para o rastreamento pela mamografia ainda é debatida na comunidade científica. A realização do exame em mulheres na faixa de 40 a 49 anos é questionada, uma vez que, nessa faixa, observa-se um aumento no número de resultados falsopositivos. A incidência de câncer de mama nas mulheres dessa faixa etária é menor do que em mulheres com 50 anos ou mais (8, 9). A Sociedade Brasileira de Mastologia (SBM) defende que o exame seja iniciado nas mulheres com 40 anos e que seja realizado em intervalos de 1 ano (7, 8). Quanto ao procedimento para rastreamento de câncer de colo uterino, é indicado que o Papanicolaou seja realizado nas mulheres sexualmente ativas, prioritariamente na faixa entre 25 e 60 anos. Um intervalo de 3 anos entre exames é recomendado apenas nas mulheres com dois exames negativos no prazo de 1 ano (1, 9).

O objetivo deste artigo é propor um indicador para classificar a condição preventiva das mulheres como desejável, alerta e de risco considerando a combinação dos exames de mamografia e Papanicolaou e considerando as indicações de realização desses exames para cada faixa etária.

## INDICADOR PREVENTIVO DE SAÚDE DA MULHER

Há muitos estudos disponíveis acerca dos benefícios, cobertura e realização da mamografia e do Papanicolaou (1, 2, 7–13). Contudo, tais estudos analisam os dois tipos de câncer de forma isolada e independente, apesar das evidências de forte relação entre a realização de ambos, já que possuem o mesmo médico solicitante (7, 10, 16). Tendo como base as recomendações para mamografia e Papanicolaou, pode-se propor a criação de um indicador que combine os dois exames para expressar de forma gradual o quão adequado é o status de prevenção de cada mulher. Ato contínuo, como corolário em um contexto populacional, é possível quantificar a proporção de mulheres com status preventivo adequado para esses dois cânceres, redundando em um indicador que poderia ser reconhecido como indicador preventivo de saúde da mulher (IPSM). Com tal indicador, além da quantificação da condição de prevenção na população feminina, seriam possíveis comparações entre características e condições de interesse (tais como idade, região ou etnia), acompanhamento de variações temporais e avaliação de melhorias nas condições de saúde e políticas públicas dirigidas à população feminina. A identificação e o uso de indicadores que gozem de validade, confiabilidade e sensibilidade para detectar variações é sempre um desafio em saúde pública.

Basicamente, o IPSM propõe considerar o período decorrido desde a última realização dos dois exames e também a idade da mulher, apresentando três condições em uma escala ordinal, com a seguinte construção: a condição “desejável” ou “ideal” é representada pela cor verde, indicando que a mulher encontra-se em nível considerado ideal de prevenção; o segundo nível é representado pela cor amarela, expressando uma condição de alerta; e o terceiro nível indica uma condição de risco (cor vermelha), ou seja, inadequação na realização dos dois exames preventivos. Adicionalmente, propõe-se que os tempos decorridos desde a realização da última mamografia e do último Papanicolaou sejam classificados em três categorias para cada exame: mamografia realizada 1) há menos de 2 anos; 2) há 2 anos ou mais; e 3) nunca realizada; e Papanicolaou realizado há 1) menos de 3 anos; 2) há 3 anos ou mais; e 3) nunca realizado.

Para completar a ideia, propõe-se que o ISPM considere as realizações dos exames especificamente de acordo com cinco faixas etárias, combinando as indicações do Ministério da Saúde e da SBM: 1) 25 a 40 anos; 2) 41 a 50 anos; 3) 51 a 60 anos; 4) 61 a 70 anos; e 5) 71 anos ou mais. A primeira faixa representa a idade na qual o foco, de modo geral, está voltado apenas para a realização do Papanicolaou; podendo, inclusive, ser estendida para idades mais precoces, como 18 anos. Entre 41 e 50 anos, enfocam-se mulheres que, além da realização de Papanicolaou, já têm indicação segundo a SBM para rastreamento de câncer de mama pela mamografia. Já a faixa entre 51 e 60 anos abrange a idade inicial do rastreamento por mamografia de acordo com o Ministério da Saúde. Adicionalmente, dadas as características das mulheres idosas, essas foram separadas entre 61 e 70 anos e com 71 anos ou mais.

A tabela 1 ilustra e sintetiza a proposta do IPSM, mostrando a distribuição dos critérios para classificação na condição de risco (vermelha), desejável (verde) ou intermediária, de alerta (amarelo).

## EXEMPLO DE APLICAÇÃO

Tomando como exemplo de aplicação os dados do suplemento de saúde da Pesquisa Nacional por Amostra de Domicílios de 2008 (14), no qual foram investigadas as condições de prevenção das mulheres com 25 anos de idade ou mais, projetou-se o ISMP para o Brasil em 2008. Esse indicador mostrou que 50% das mulheres brasileiras estavam em condição desejável, enquanto 24,8% se encontravam em condição de risco e 25,2% estavam na categoria de alerta.

**FIGURA 1.**
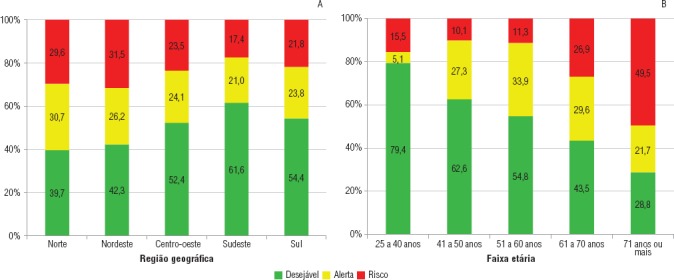
Status das mulheres brasileiras por região geográfica e faixa etária conforme o Indicador Preventivo de Saúde da Mulher aplicado a dados da Pesquisa Nacional por Amostra de Domicílios (PNAD), 2008

Também a título de ilustração, usando a mesma base de dados da PNAD, foram projetadas as faixas etárias de interesse para mamografia e Papanicolaou (Figura 1A). O exercício mostrou que no Brasil, em 2008, 5,1% das mulheres com menos de 40 anos já estavam em condição de alerta, enquanto que para as mulheres com 41 anos ou mais esse patamar variou de 21,7% a 33,9% nessa mesma condição. Das mulheres com 71 anos ou mais, 49,5% se encontravam em condição de risco, um percentual bastante mais acentuado do que o do grupo de mulheres com até 70 anos de idade. Percebe-se que a prevalência da condição desejável decresce conforme as faixas etárias aumentam e que o inverso ocorre na condição de risco.

Como tem-se relatado que ambos os cânceres apresentam distribuição de incidência e mortalidade distintas entre as regiões brasileiras (15–17), o IPSM pode ser útil para comparar estratégias de prevenção contra os cânceres de mama e colo uterino e entender as variações regionais observadas. A figura 1B mostra que as regiões diferiram bastante, com destaque positivo para a região Sudeste e negativo para as regiões Norte e Nordeste: a condição desejável foi menor do que 50% nas regiões Norte e Nordeste e a região Sudeste teve a menor prevalência da condição de risco.

**TABELA 1:** Classificação do status da mulher de acordo com o Indicador de Prevenção de Saúde da Mulher

Idade	Última mamografia	Último Papanicolaou		
		Menos de 3 anos	3 anos ou mais	Nunca fez
25 a 40 anos	Menos de 2 anos	Desejável	Alerta	Risco
	Há 2 anos ou mais	Desejável	Alerta	Risco
	Nunca fez	Desejável	Alerta	Risco
41 a 50 anos	Menos de 2 anos	Desejável	Alerta	Risco
	Há 2 anos ou mais	Desejável	Alerta	Risco
	Nunca fez	Alerta	Alerta	Risco
51 a 60 anos	Menos de 2 anos	Desejável	Alerta	Risco
	Há 2 anos ou mais	Alerta	Alerta	Risco
	Nunca fez	Alerta	Alerta	Risco
61 a 70 anos	Menos de 2 anos	Desejável	Alerta	Risco
	Há 2 anos ou mais	Alerta	Alerta	Risco
	Nunca fez	Alerta	Risco	Risco
71 anos ou mais	Menos de 2 anos	Desejável	Alerta	Risco
	Há 2 anos ou mais	Alerta	Alerta	Risco
	Nunca fez	Risco	Risco	Risco

## DISCUSSÃO

O monitoramento de agravos à saúde, especialmente em subpopulações específicas, demanda a construção de indicadores sensíveis e úteis às políticas de intervenção, como o IPSM. Em função da elevada incidência e mortalidade dos cânceres de mama e colo do útero, e tendo em vista o importante papel da mamografia e do Papanicolaou como meios preventivos, é necessário que os órgãos públicos e a comunidade científica realizem avaliações rotineiras das taxas de realização desses exames na população feminina.

É importante salientar que a validade de indicadores como o IPSM depende da qualidade da informação prestada. Portanto, dados obtidos a partir de informações autorrelatadas, como é o caso da PNAD, por exemplo, poderiam ser questionados quanto à sua validade. No entanto, como esses exames demandam cuidados e envolvimento pessoal de cada mulher, acredita-se que, caso o Idade Última mamografia Menos de 3 anos 3 anos ou mais Nunca fez indicador venha a ser utilizado em estudos futuros, as respostas fornecidas teriam boa validade. Antes disso, porém, é necessário um estudo para validar o IPSM.

Uma limitação deste indicador, em virtude da pesquisa utilizada para construí-lo, é que ele não capta as mulheres que tiveram um exame positivo e que, porventura, tenham a recomendação de realização desses exames em prazo menor do que o indicado para a faixa etária. Uma forma de reduzir esse viés seria analisar separadamente este subgrupo de mulheres que tiveram o exame positivo e aplicar o indicador para as novas faixas de recomendação.

O IPSM poderá vir a ser utilizado como um instrumento de avaliação das ações de saúde, uma vez que permite, de forma simples, o estudo simultâneo da frequência com que as mulheres realizam tanto a mamografia quanto o Papanicolaou. Cabe ressaltar que esse indicador foi construído levando em conta as recomendações para cada faixa etária no Brasil, de modo que não é imediata a extrapolação ou comparação para outros países. Possui pronta aplicação nas comparações preventivas ao longo do tempo e em diferenças interregionais de um mesmo país, de modo a reconhecer efeitos de eventuais políticas de saúde preventivas para a mulher. Entretanto, se fosse a intenção de outro país incorporar o IPSM, algumas adaptações seriam necessárias. Estudos futuros devem ser realizados para validar e desenvolver o IPSM em diversos contextos.

### Agradecimentos.

Este projeto foi parcialmente financiado pela Fundação de Amparo à Pesquisa do Estado do Rio de Janeiro (FAPERJ) (processo n. E-26/102.357/2013 e E-26/224.838/2016).

### Declaração de responsabilidade.

A responsabilidade pelas opiniões expressas neste manuscrito é estritamente dos autores e não reflete necessariamente as opiniões ou políticas da RPSP/PAJPH nem da OPAS.
